# Circulating thrombospondin 2 levels reflect fibrosis severity and disease activity in HCV-infected patients

**DOI:** 10.1038/s41598-022-23357-9

**Published:** 2022-11-07

**Authors:** Takanobu Iwadare, Takefumi Kimura, Naoki Tanaka, Tomoo Yamazaki, Shun-ichi Wakabayashi, Taiki Okumura, Hiroyuki Kobayashi, Yuki Yamashita, Sai P. Pydi, Tomoyuki Nakajima, Mai Iwaya, Ayumi Sugiura, Satoru Joshita, Takeshi Uehara, Takeji Umemura

**Affiliations:** 1 Department of Medicine, Division of Gastroenterology and Hepatology, Shinshu University School of Medicine, 3-1-1 Asahi, Matsumoto, Nagano 390-8621 Japan; 2 Consultation Center for Liver Diseases, Shinshu University Hospital, Matsumoto, Japan; 3 International Relations Office, Shinshu University School of Medicine, Matsumoto, Japan; 4 Department of Metabolic Regulation, Shinshu University School of Medicine, Matsumoto, Japan; 5 Department of Medicine, University of California San Diego, La Jolla, CA USA; 6 Department of Biological Sciences and Bioengineering, Indian Institute of Technology, Kanpur, India; 7 Department of Laboratory Medicine, Shinshu University School of Medicine, Matsumoto, Japan

**Keywords:** Biomarkers, Diseases, Gastroenterology

## Abstract

Among several secreted glycoproteins belonging to the thrombospondin family, thrombospondin 2 (TSP2) is involved in various functions, including collagen/fibrin formation. Liver/serum TSP2 levels have been correlated to liver fibrosis stage and disease activity in nonalcoholic fatty liver disease. This study investigated whether serum TSP2 was associated with clinicopathological features in hepatitis C virus (HCV)-infected patients as well. A total of 350 patients with HCV who had undergone liver biopsy were retrospectively enrolled and divided into a discovery cohort (n = 270) and a validation cohort (n = 80). In the discovery cohort, serum TSP2 levels were moderately correlated with both liver fibrosis stage (*r* = 0.426, P < 0.0001) and activity grade (*r* = 0.435, P < 0.0001). The area under the receiver operating characteristic curve of TSP2 for predicting severe fibrosis (≥ F3) was 0.78 and comparable to or better than those of autotaxin (0.78), FIB-4 index (0.78), and APRI (0.76). The discovery cohort findings were closely replicated in the validation cohort. Moreover, comprehensive liver genetic analysis of HCV-infected patients confirmed that the expression of the *THBS2* gene encoding TSP2 was significantly higher in severely fibrotic F4 than in F1 patients. Circulating TSP2 levels may reflect the severity of hepatic fibrosis/inflammation in HCV-infected patients.

## Introduction

Hepatitis C virus (HCV) infection is the leading cause of chronic liver disease, with approximately 71 million chronically infected people worldwide^1^. HCV infection is also known as a major risk factor for hepatocellular carcinoma (HCC) development^2–4^. Chronic inflammation and cytokine release caused by HCV lead to fibrosis and hepatocyte proliferation, which are considered the main pathogenic mechanisms of HCC^5,6^. While many HCV patients have benefited from revolutionary direct-acting antiviral (DAA) treatment towards viral elimination^7^, the prohibitively high cost of DAAs has left many HCV patients without access to treatment worldwide^8,9^. As long as HCV cannot be eliminated completely, efficiently identifying infected patients at more advanced disease stages remains an important challenge.

The thrombospondin 2 (TSP2) protein encoded by the *THBS2* gene is involved in collagen/fibrin formation, bone growth, maintenance of normal vessel density, hemostasis, and cell adhesion^10^. We earlier demonstrated TSP2 as a potential serum biomarker for clinical application in nonalcoholic fatty liver disease (NAFLD)/nonalcoholic steatohepatitis due to its strong correlations with hepatocellular ballooning, NAFLD activity score, and fibrosis stage in biopsy-proven NAFLD cases^11^. These results have since been replicated in multiple studies using a comprehensive genetic analysis approach in the livers of NAFLD patients and in a large study of NAFLD patients with diabetes mellitus^12,13^. However, the role of TSP2 in viral hepatitis remains unknown. This study evaluated the utility of serum TSP2 levels as a clinicopathological indicator in HCV-infected patients.

## Results

### Clinicopathological features of HCV-infected patients in the discovery cohort

The clinicopathological features of the 270 HCV-infected patients in the discovery cohort are presented in Table 1. Median age was 60 years, and 131 patients (49%) were male. Liver enzymes including aspartate aminotransferase (AST), alanine aminotransferase (ALT), alkaline phosphatase (ALP), and gamma-glutamyl transpeptidase (GGTP) showed levels slightly above normal values. Median serum TSP2 was 38.9 ng/mL (IQR: interquartile range 28.2–54.4). The median values of such conventional liver-fibrotic parameters as autoaxin (ATX), fibrosis-4 index (FIB-4 index), Forn’s index, and AST to platelet ratio index (APRI) were 1.5, 2.63, 6.02, and 1.3, respectively. HCV genotypes 1 and 2 totaled 181 and 89 cases, respectively. The respective number of patients with fibrosis stage F0–1, F2, and F3–4 was 121, 66, and 83. The number of patients exhibiting activity grade A0, A1, A2, and A3 was 25, 96, 134, and 15, respectively.

**Table 1:** Clinicopathological features of HCV-infected patients in the discovery cohort.

	All (N = 270)
	Median (IQR)/N (%)
Age (years)	60 (51–66)
Male	131 (49%)
Body mass index (kg/m^2^)	23.7 (21.2–25.7)
**Laboratory data**	
Albumin (mg/dL)	4.4 (4.1–4.5)
Total bilirubin (mg/dL)	0.80 (0.63–1.00)
Direct bilirubin (mg/dL)	0.16 (0.10–0.22)
AST (U/L)	52 (37–89)
ALT (U/L)	70 (46–113)
ALP (U/L)	289 (234–367)
GGTP (U/L)	42 (25–79)
Total cholesterol (mg/dL)	175 (154–199)
Triglycerides (mg/dL)	96 (70–135)
Alpha-fetoprotein (ng/mL)	5.45 (3.3–10.6)
PT (%)	96.8 (86.6–105.1)
TSP2 (ng/mL)	38.9 (28.2–54.4)
ATX (mg/L)	1.5 (1.0–2.1)
FIB-4 index	2.63 (1.61–3.85)
Forn’s index	6.02 (4.56–7.24)
APRI	1.3 (0.7–2.3)
Platelet count (× 10^4^/µL)	15.7 (12.3–19.5)
**HCV**	
Genotype (1/2)	181/89
**Pathology**	
METAVIR	
Fibrosis stage (F0–1/F2/F3–4)	121/66/83
Activity grade (A0/A1/A2/A3)	25/96/134/15

*ALT* alanine aminotransferase, *ALP* alkaline phosphatase, *APRI* aspartate aminotransferase to platelet ratio index, *AST* aspartate aminotransferase, *ATX* autotaxin, *FIB-4 index* fibrosis-4 index, *GGTP* gamma-glutamyl transpeptidase, *HCV* hepatitis C virus, *IQR* interquartile range, *PT* prothrombin time, *TSP2* thrombospondin 2.

### Correlation between serum TSP2 levels and clinical features of HCV-infected patients in the discovery cohort

Table 2 summarizes the correlation analysis of clinical parameters with serum TSP2 levels. We observed weak correlations between TSP2 and albumin (*r* = − 0.219, P = 0.0004), direct bilirubin (*r* = 0.247, P = 0.0009), ALP (*r* = 0.288, P < 0.0001), prothrombin time (*r* = − 0.211, P = 0.0033), platelet count (*r* = − 0.284, P < 0.0001), and Forn’s index (*r* = 0.353, P < 0.0001), with none for age, body mass index, total bilirubin, or lipid profiles including total cholesterol and triglycerides. TSP2 was moderately correlated to the hepatic parameters of AST (*r* = 0.453, P < 0.0001), ALT (*r* = 0.412, P < 0.0001), GGTP (*r* = 0.414, P < 0.0001), alpha-fetoprotein (*r* = 0.469, P < 0.0001), ATX (*r* = 0.556, P < 0.0001), FIB-4 index (*r* = 0.402, P < 0.0001), and APRI (*r* = 0.433, P < 0.0001). Interestingly, moderate correlations of TSP2 were found with fibrosis grade (*r* = 0.426, P < 0.0001) and activity grade (*r* = 0.435, P < 0.0001) among pathological findings.

**Table 2:** Correlation between TSP2 and clinicopathological parameters in the discovery cohort.

	Correlation with TSP2 (N = 270)	
	*r*	P value
Age	0.023	0.7095
Body mass index	0.086	0.2867
**Laboratory data**		
Albumin	− 0.219	**0.0004**
Total bilirubin	0.008	0.8965
Direct bilirubin	0.247	**0.0009**
AST	0.453	** < 0.0001**
ALT	0.412	** < 0.0001**
ALP	0.288	** < 0.0001**
GGTP	0.414	** < 0.0001**
Total cholesterol	− 0.127	0.0597
Triglycerides	0.109	0.1251
Alpha-fetoprotein	0.469	** < 0.0001**
PT	− 0.211	**0.0033**
ATX	0.556	** < 0.0001**
FIB-4 index	0.402	** < 0.0001**
Forn’s index	0.353	** < 0.0001**
APRI	0.433	** < 0.0001**
Platelet count	− 0.284	** < 0.0001**
**Pathology**		
METAVIR		
Fibrosis stage	0.426	** < 0.0001**
Activity grade	0.435	** < 0.0001**

Significant values are in bold.

Correlations were calculated using Spearman’s test.

*ALT* alanine aminotransferase, *ALP* alkaline phosphatase, *APRI* aspartate aminotransferase to platelet ratio index, *AST* aspartate aminotransferase, *ATX* autotaxin, *FIB-4 index* fibrosis-4 index, *GGTP* gamma-glutamyl transpeptidase, *PT* prothrombin time, *TSP2* thrombospondin 2.

### Performance of serum TSP2 levels for estimating fibrosis stage and activity grade of HCV-infected patients in the discovery cohort

**Figure 1:**
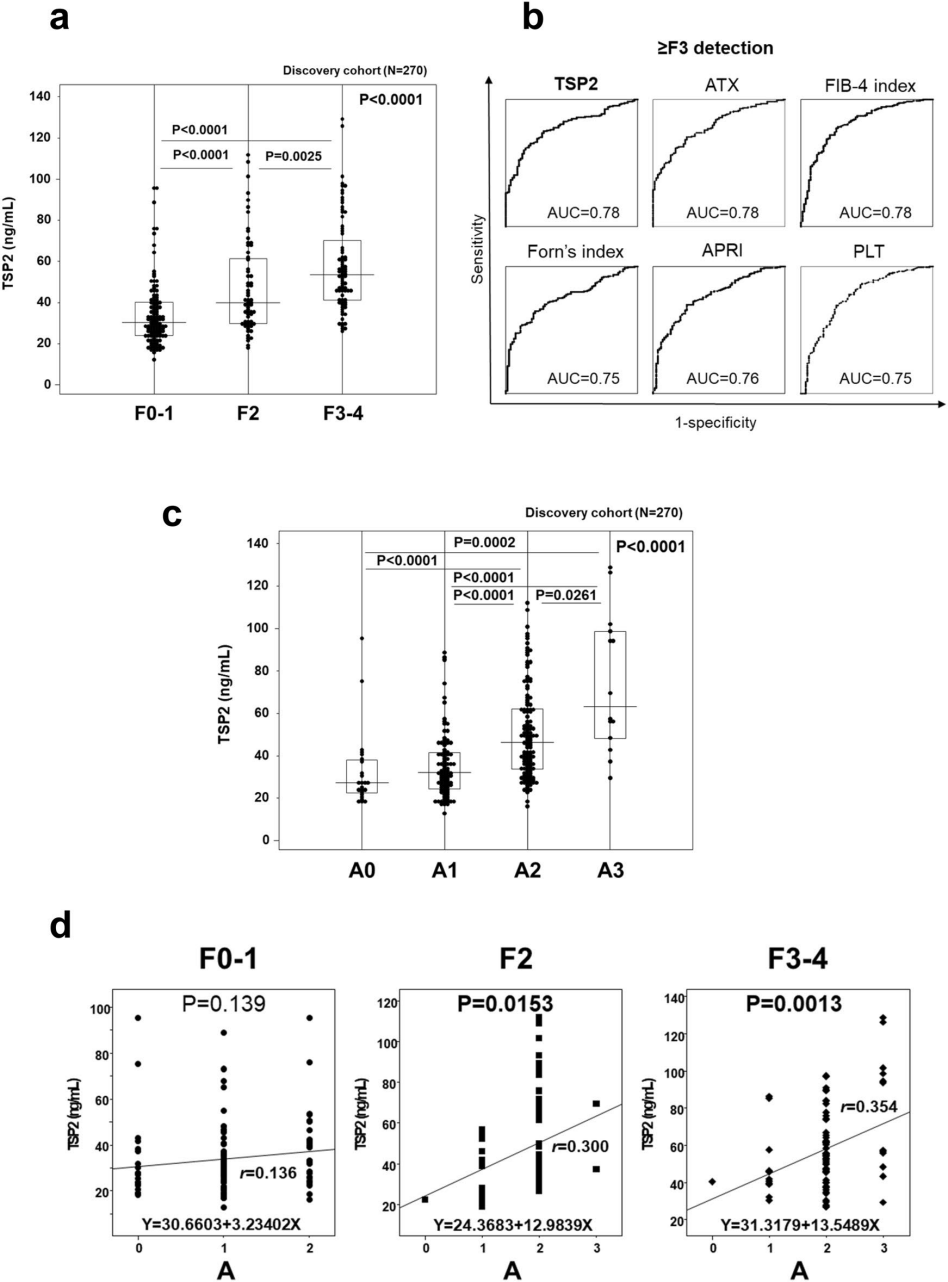
Positive correlation between serum TSP2 levels and fibrosis stage/activity grade in HCV-infected patients in the discovery cohort. (**a**) Correlation of serum TSP2 levels and fibrosis stage. (**b**) AUC values for estimating severe fibrosis stage (≥ F3). (**c**) Correlation of serum TSP2 levels and activity grade. (**d**) Correlation of serum TSP2 levels and activity grade at different fibrosis stages. Data were obtained from 270 cases in the discovery cohort. *A* activity grade, *APRI* aspartate aminotransferase to platelet ratio index, *ATX* autotaxin, *AUC* area under the receiver operating characteristic curve, *F* fibrosis stage, *FIB-4 index* fibrosis-4 index, *PLT* platelet count, *TSP2* thrombospondin 2.

The relationship between serum TSP2 levels and pathological indicators was addressed next. Similarly to findings in NAFLD^11^, TSP2 levels were significantly increased in patients with more advanced liver fibrosis (Fig. 1a). The area under the receiver operating characteristic curve (AUC) in predicting severe fibrosis (≥ F3) was 0.78 for TSP2, 0.78 for ATX, 0.78 for FIB-4 index, 0.75 for Forn index, 0.76 for APRI and 0.75 for platelet count (Fig. 1b). Comparative analysis of AUCs by the Delong method showed that TSP2 and other indicators were statistically comparable (vs. ATX: P = 0.800, vs. FIB-4 index: P = 0.546, vs. Forn’s index: P = 0.307, vs. APRI: P = 0.466, and vs. platelet count: P = 0.804).The sensitivity, specificity, positive predictive value (PPV), and negative predictive value (NPV) for predicting advanced fibrosis stage (≥ F3) at the TSP2 cut-off value (45.3 ng/mL) were 75.1%, 71.3%, 85.8%, and 55.3%, respectively (Table 3).

**Table 3:** Diagnostic performance of TSP2 and other clinical parameters for predicting advanced fibrosis stage (≥ F3) and activity grade (≥ A2) in patients with HCV in the discovery cohort.

	Cut-off value	AUC	Sensitivity (%)	Specificity (%)	PPV (%)	NPV (%)
**≥ F3**						
TSP2	45.3 (ng/mL)	0.78	75.1	71.3	85.8	55.3
ATX	1.68 (mg/L)	0.78	70.5	74.3	86.0	53.0
FIB-4 index	3.0	0.78	72.0	75.6	86.0	54.4
Forn’s index	6.2	0.75	66.5	72.8	84.8	48.8
APRI	1.57	0.76	70.4	69.5	83.9	50.9
Platelet count	14.6 (× 10^4^/µL)	0.75	71.5	69.5	84.2	51.8
**≥ A2**						
TSP2	39.5 (ng/mL)	0.75	70.6	64.4	61.8	72.9
ATX	1.69 (mg/L)	0.74	77.3	59.6	60.9	76.3
FIB-4 index	2.8	0.70	68.6	62.0	59.7	70.5
Forn’s index	6.4	0.65	69.2	53.5	54.9	67.8
APRI	1.5	0.75	78.5	62.6	63.5	78.6
Platelet count	14.8 (× 10^4^/µL)	0.64	67.8	52.4	53.9	66.4

*APRI* aspartate aminotransferase to platelet ratio index, *ATX* autotaxin, *AUC* area under the receiver operating characteristic curve, *NPV* negative predictive value, *PPV* positive predictive value, *FIB-4 index* fibrosis-4 index, *TSP2* thrombospondin 2.

TSP2 levels were also significantly increased in patients with more advanced activity grade (Fig. 1c). Correlation analysis of activity grade with serum TSP2 levels according to fibrosis stage revealed weak correlations in F2 patients (*r* = 0.300, P = 0.0153) and F3-4 patients (*r* = 0.354, P = 0.0013), with none in F0-1 patients (Fig. 1d). The TSP2 cut-off value (39.5 ng/mL) for predicting moderate-severe activity grade (≥ A2) showed the highest AUC among tested parameters, providing sensitivity, specificity, PPV, and NPV findings of 70.6%, 64.4%, 61.8%, and 72.9%, respectively (Table 3). AUC comparison analysis by the Delong method showed that the AUC of TSP2 was statistically higher than AUC of Forn index (P = 0.023) and similar to AUC of ATX (P = 0.784), FIB-4 index (P = 0.136), APRI (P = 0.997), platelet count (P = 0.204).

### Relationship between serum TSP2 levels and clinical features of patients with HCV in the validation cohort

The clinicopathological features of the 80 HCV-infected patients enrolled in the validation cohort are listed in Supplementary Table 1. Correlation analysis of clinical parameters with serum TSP2 levels in Supplementary Table 2 uncovered weak correlations between TSP2 and albumin (*r* = − 0.274, P = 0.0168), ALP (*r* = 0.337, P = 0.0029), FIB-4 index (*r* = 0.374, P = 0.0007), Forn’s index (*r* = 0.379, P = 0.0007), and platelet count (*r* = − 0.357, P = 0.0014). TSP2 was moderately correlated to the hepatic parameters of direct bilirubin (*r* = 0.404, P = 0.0048), AST (*r* = 0.488, P < 0.0001), ALT (*r* = 0.479, P < 0.0001), GGTP (*r* = 0.521, P < 0.0001), alpha-fetoprotein (*r* = 0.657, P < 0.0001), ATX (*r* = 0.511, P < 0.0001), and APRI (*r* = 0.460, P < 0.0001). Regarding pathological findings, TSP2 was moderately correlated to fibrosis stage (*r* = 0.465, P = 0.0001) and activity grade (*r* = 0.449, P = 0.0003). The above validation cohort data were in strong agreement with the results of the discovery cohort.

### Performance of serum TSP2 levels for estimating fibrosis stage and activity grade of HCV-infected patients in the validation cohort

**Figure 2:**
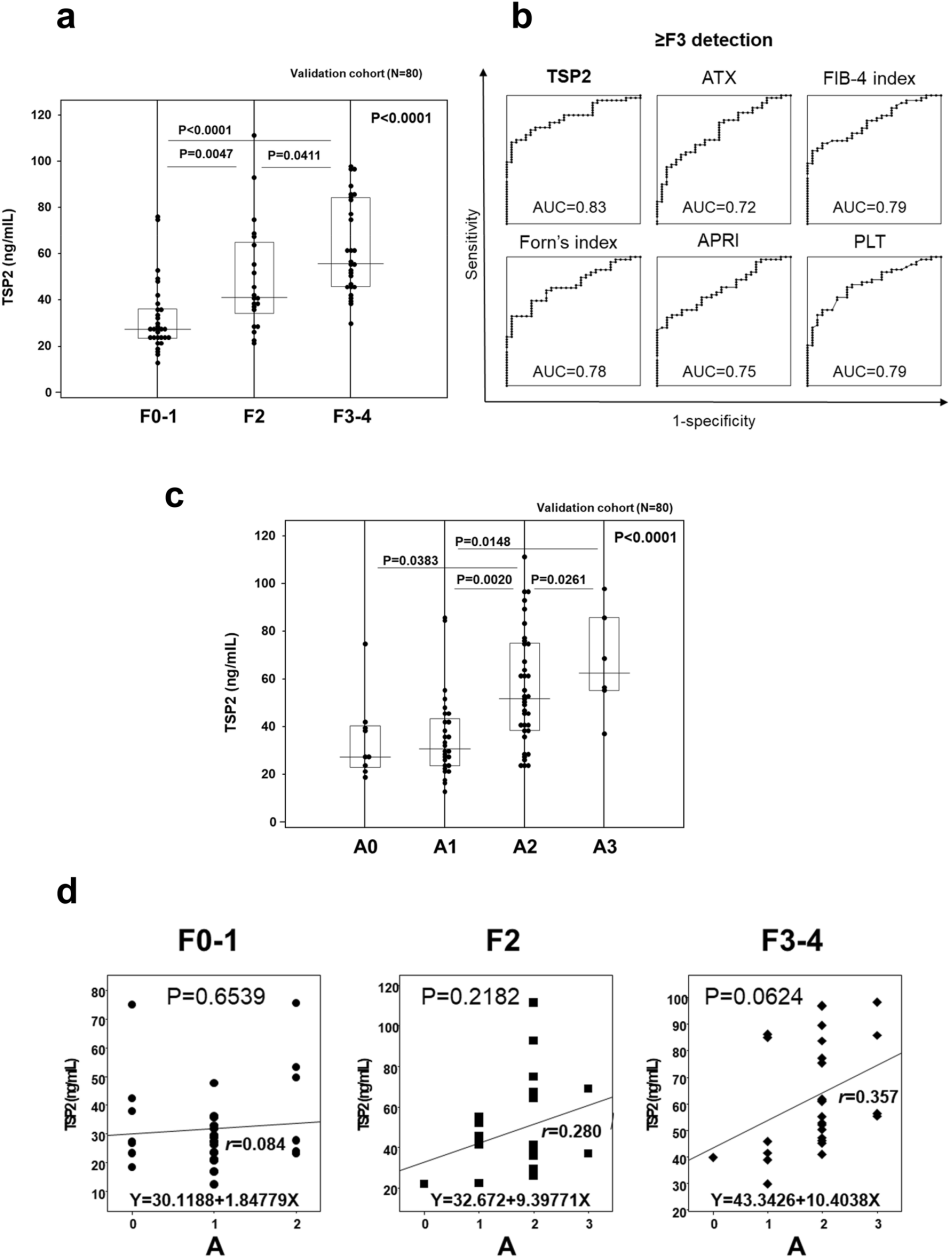
Validation of correlation between serum TSP2 levels and fibrosis stage/activity grade in HCV-infected patients. (**a**) Correlation of serum TSP2 levels and fibrosis stage. (**b**) AUC values for estimating severe fibrosis stage (≥ F3). (**c**) Correlation of serum TSP2 levels and activity grade. (**d**) Correlation of serum TSP2 levels and activity grade at different fibrosis stages. Data were obtained from 80 cases in the validation cohort. *A* activity grade, *APRI* aspartate aminotransferase to platelet ratio index, *ATX* autotaxin, *AUC* area under the receiver operating characteristic curve, *F* fibrosis stage, *FIB-4 index* fibrosis-4 index, *PLT* platelet count, *TSP2* thrombospondin 2.

TSP2 levels were significantly increased in patients with more advanced liver fibrosis in the validation cohort (Fig. 2a). The AUC of TSP2 for predicting severe fibrosis (≥ F3) was 0.83 and greater than those of ATX (0.72), FIB-4 index (0.79), Forn’s index (0.78), APRI (0.75), and platelet count (0.79) (Fig. 2b). Comparative analysis of AUCs by the Delong method showed that TSP2 and other indicators were statistically comparable (vs. ATX: P = 0.189, vs. FIB-4 index: P = 0.477, vs. Forn index: P = 0.390, vs. APRI: P = 0.184, and vs. platelet count: P = 0.780). A TSP2 cut-off value (50.3 ng/mL) for predicting severe fibrosis (≥ F3) provided the highest AUC among tested parameters, with sensitivity, specificity, PPV, and NPV of 78.8%, 68.0%, 82.0%, and 63.3%, respectively (Supplementary Table 3).

TSP2 levels were also increased in patients with more advanced activity grade (Fig. 2c). Correlation analysis of activity grade with serum TSP2 levels based on fibrosis stage showed a trend towards correlation in F2 and F3–4 patients, although this difference did not reach statistical significance (P = 0.2182 and P = 0.0624, respectively) (Fig. 2d). A TSP2 cut-off value (47.8 ng/mL) for predicting moderate-severe activity grade (≥ A2) showed the highest AUC among tested parameters, with respective sensitivity, specificity, PPV, and NPV of 83.8%, 60.5%, 64.5%, and 81.2% (Supplementary Table 3). AUC comparison analysis by the Delong method showed that the AUC of TSP2 was statistically similar to AUC of ATX (P = 0.121), FIB-4 index (P = 0.136), Forn index (P = 0.085), APRI (P = 0.361), platelet count (P = 0.243).

Taken together, the validation data confirmed the remarkable correlations of serum TSP2 with fibrosis stage and activity grade observed in the discovery cohort.

### Liver mRNA analysis of HCV-infected patients

**Figure 3:**
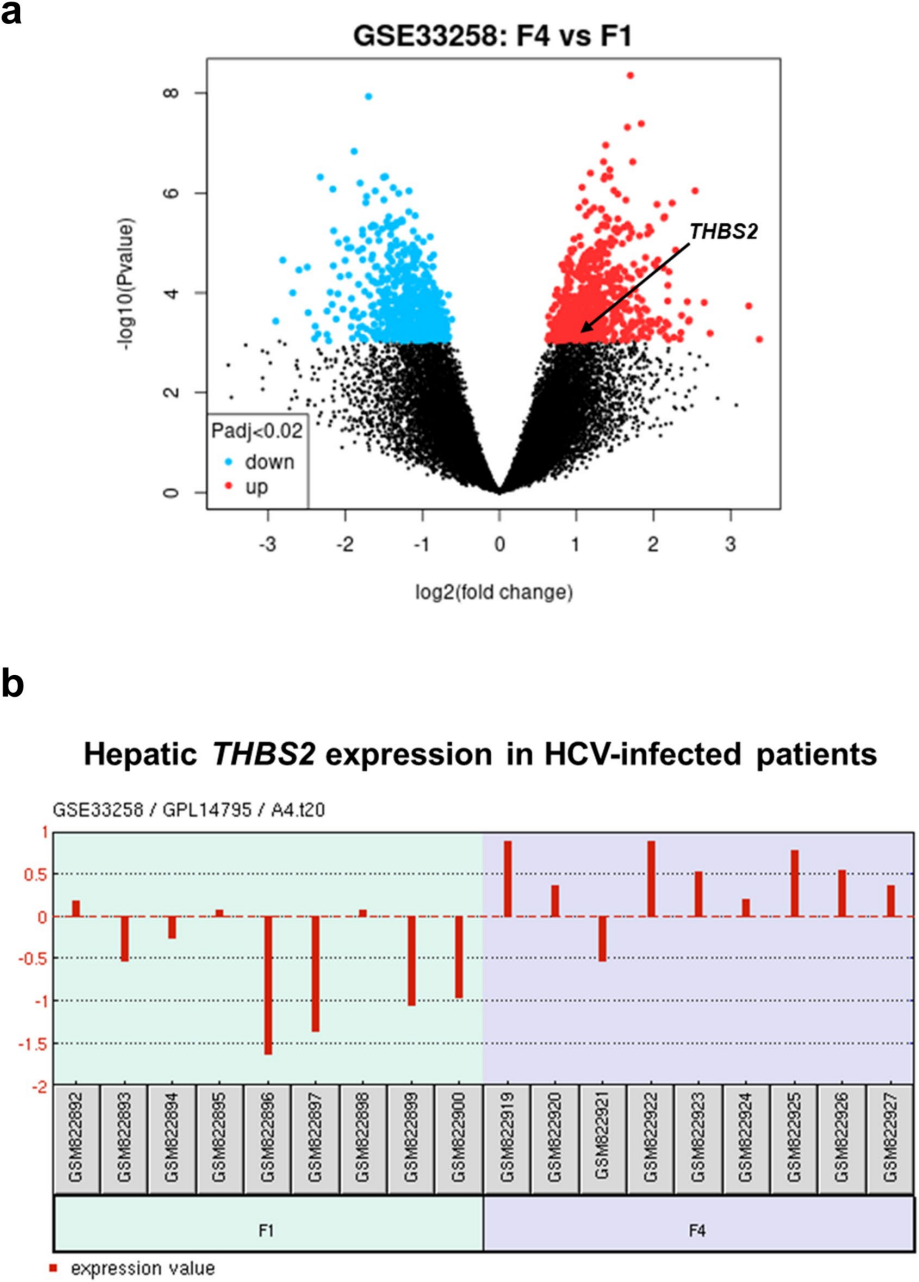
Liver mRNA data analysis of HCV-infected patients using GSE33258. (**a**) Volcano plot extracted from GSE33258. This plot visualizes differentially expressed genes by displaying statistical significance (− log10 P value) and magnitude of change (log2FC). Genes are significantly differentially expressed with a P value cut-off of 0.02 (red = up-regulated, blue = down-regulated). Arrows indicate *THBS2*, the gene responsible for TSP2. (**b**) THBS2 expression profiles for each case included in GSE33258. *F* fibrosis stage, *HCV* hepatitis C virus.

The hepatic expression levels of the *THBS2* gene responsible for TSP2 were examined next. mRNA expression data for fibrosis stage in HCV-infected patients were extracted from the microarray data set (GSE33258) and analyzed^14^. F4 stage livers showed more up- and down-regulation of mRNA genes compared with F1 stage livers. *THBS2* was included among the 853 genes meeting the threshold of adjusted P < 0.02 and |log2FC| > 1 as genes with large changes (Fig. 3a). The log2FC of *THBS2* was 1.08, with an adjusted P = 0.018628. Elevated mRNA expression levels of *THBS2* were more frequent in F4 cases than in F1 cases (Fig. 3b). Considering the nature of TSP2 as a secreted protein, TSP2 overproduction in the liver may leak into the serum to account for the correlation between serum TSP2 and liver fibrosis.

## Discussion

This study identified several relationships between the serum TSP2 levels of HCV-infected patients and pathological indicators (Supplementary Fig. 1). Similar to a study of patients with NAFLD, serum TSP2 was moderately correlated with fibrosis stage in HCV infection (*r* = 0.426, P < 0.0001). TSP2 had an AUC of 0.78 for predicting severe fibrosis (≥ F3), which was comparable to or better than those of ATX, FIB-4 index, Forn’s index, and APRI. Furthermore, liver transcriptomic data from HCV-infected patients showed that *THBS2* gene expression was higher in F4 cases than in F1 cases. These data substantiate the hypothesis that increased liver TSP2 production in HCV-infected patients with advanced fibrosis leads to elevated serum TSP2. Interestingly, TSP2 exhibited a moderate association with activity grade (*r* = 0.435, P < 0.0001) that was especially pronounced in fibrosis stages F2 and F3–4. These findings suggest that TSP2 may be a useful marker to estimate the severity of hepatic fibrosis and inflammation in HCV-infected patients. To our knowledge, this is the first report of serum TSP2 being associated with clinicopathological indicators in HCV.

In chronic hepatitis C, noninvasive alternatives to liver biopsy are preferable to assess the severity of liver damage before treatment. Serum biomarkers for estimating fibrosis can be divided into direct and indirect forms. Direct biomarkers are collagen fibers produced during extracellular matrix remodeling via activated hematopoietic stem cells, hyaluronic acid, YKL-40, laminin, fibronectin, metalloproteinases, tissue inhibitors of metalloproteinases, and such extracellular matrix (ECM) metabolites as transforming growth factor-β1 (TGF-β)^15^. Indirect biomarkers biochemically analyze serum and patient clinical parameters, such as AST-ALT ratio, APRI, FIB-4 index, and Forn’s index^16,17^. ATX is metabolized by hepatic sinusoidal endothelial cells and has been associated with liver injury^18^. We recently reported that serum ATX had diagnostic value for liver fibrosis in hepatitis C patients^19^. The present study demonstrated that serum TSP2 levels exhibited an ability to identify F3-4 advanced fibrosis comparably to or better than the established fibrosis markers of ATX, FIB-4 index, Forn's index, and APRI.

Thrombospondins are calcium-binding glycoproteins that interact with other ECM components^20^. They have properties in common with other matrix molecules, cytokines, adaptor proteins, and chaperones, regulate collagen fiber organization, and bind and localize a variety of growth factors and proteases^21^. The interactions of thrombospondins with different receptors on the cell surface evoke cell-dependent signaling and phenotypic changes that enhance wound healing, angiogenesis, vessel wall biology, connective tissue formation, and synapse formation^20,21^. While basic studies on the function of TSP2 in the liver are very limited, Lindert et al. reported that in primary rat hepatic stellate cells, the induction of collagen type I and TSP2 was stimulated by both Smad-dependent and MAPK-dependent TGF-β signaling^22^. Given that TSP2 is activated by TGF-β signaling during the progression of liver fibrosis, it is plausible that TSP2 is upregulated in the liver and secreted into the blood during fibrosis exacerbation in HCV-infected patients. Our previous study showed that serum TSP2 closely reflected fibrosis in patients with NAFLD^11^. An important message from the present investigation of HCV-infected patients is that the correlation between liver fibrosis and TSP2 may not be specific to NAFLD, but rather a universal event in chronic liver disease.

Our earlier report on TSP2 in NAFLD also revealed correlations not only with liver fibrosis, but also with hepatocyte ballooning and inflammation^11^. It was very suggestive in the present study of HCV-infected patients that TSP2 levels were also associated with activity grade independently of fibrosis stage. The fact that TSP2 correlated with disease activity indicated that it retained a mechanism related to fibrosis as well as hepatic inflammation. Indeed, TSP2 is reportedly involved in inflammation during the pathogenesis of osteoarthritis by promoting interleukin-6 production in synovial fibroblasts via the PI3K/AKT/NF-κB pathway^23^. Future studies are needed to clarify the role of TSP2 in hepatitis pathogenesis.

This study had several limitations. It was retrospective, single-center, and limited in size. Since the subjects were uniformly Japanese, future studies in larger cohorts of other ethnicities are needed to validate our findings.

In conclusion, TSP2 is potentially suitable for clinical application in the field of HCV as a serum biomarker owing to its promising correlations with the severity of liver fibrosis and disease activity. Additional studies on the precise role of TSP2 in the liver are warranted.

## Methods

### Patients and clinical examinations

We retrospectively enrolled 270 biopsy-proven Japanese chronically HCV-infected patients who were admitted to Shinshu University Hospital (Matsumoto, Japan) between 2005 and 2012 as a discovery cohort. Additionally, 80 biopsy-proven Japanese chronically HCV-infected patients admitted to Shinshu University Hospital between 2013 and 2015 were retrospectively recruited as a validation cohort. The diagnosis of HCV infection was based on previously reported criteria as the presence of serum HCV antibodies and detectable HCV RNA^24^. All patients were negative for hepatitis B surface antigen as well as antibodies to hepatitis B core antigen and the human immunodeficiency virus. No patients complicated with HCC were included. Patients who were diagnosed as having alcoholic liver disease, defined as an average daily consumption of > 60 g of ethanol, were excluded. Patients with evidence of other liver disease, such as non-alcoholic liver disease, primary biliary cholangitis, or autoimmune hepatitis, were excluded as well. This study was reviewed and approved by the Institutional Review Board of Shinshu University Hospital (Matsumoto, Japan) (approval number: 3021), and written informed consent was obtained from all participating subjects. This investigation was conducted according to the principles of the Declaration of Helsinki.

Body weight and height were measured before liver biopsy in an overnight fasting condition. All laboratory data were obtained in an overnight fasting state on the day of liver biopsy. The method for measuring serum ATX was the same as previously reported^19^. FIB-4 index, Forn’s index, and APRI were calculated according to the following formulae: FIB-4 index = (age [years] × AST [IU/L])/(platelet count [10^9^/L] ×ALT [IU/L]^1/2^) ^25^, Forn’s index = 7.811 − (3.131 × ln platelet count [× 10^4^/uL]) + (0.781 × ln GGTP [U/L]) + (3.647 × ln age [years]) – (0.0114 × cholesterol [mg/dL])^26^, and APRI = (AST/upper limit of normal; 28 [U/L]) × (100/platelet count [10^9^/L])^27^. Serum TSP2 concentrations were determined using enzyme-linked immunosorbent assays (Quantikine® ELISA, #DTSP20, R&D Systems, Minneapolis, MN). Serum was obtained after overnight fasting on the day of liver biopsy and stored at – 30 °C until testing.

### Histological findings

Liver specimens of at least 1.5 cm in length were obtained from segments 5 or 8 using a 14-gauge needle as described previously and immediately fixed in 10% neutral formalin^25^. Sections of 4 μm in thickness were cut and stained using the hematoxylin and eosin and Azan-Mallory methods. The histological activity of HCV was assessed by independent expert pathologists (M.I. and T.U.) in a blinded manner according to the METAVIR scoring system^28^. Disease activity grade was scored as follows: A0, none; A1, minimal activity; A2, moderate activity; and A3, severe activity. Fibrosis stage was scored as follows: F0, none; F1, perisinusoidal or periportal; F2, perisinusoidal and portal/periportal; F3, bridging fibrosis; and F4, cirrhosis^28^.

### Statistical analysis of clinical data

Clinical data were expressed as the number (percentage) or as the median (IQR). Statistical analyses were performed using StatFlex Ver. 7.0 and Prism 8 (GraphPad). The Mann–Whitney U test was used for comparisons between the study groups. The Kruskal–Wallis test was employed for comparisons of more than two groups. Diagnostic accuracy was evaluated using the AUC. The Youden index identified cut-off values, with the nearest clinically applicable value to the cut-off considered the optimal threshold for clinical convenience. Correlation analysis was conducted using Spearman’s test. When labeling the strength of an association in this study, absolute values of *r* of 0–0.19, 0.20–0.39, and 0.40–0.59 were regarded as very weak, weak, and moderate correlations, respectively^29^. The DeLong method was used to compare the difference in the two AUCs. All statistical tests were two-sided and evaluated at the 0.05 level of significance.

### Liver mRNA data collection and processing

The mRNA expression data from the Gene Expression Omnibus (GEO) database was downloaded through a microarray data set (GSE33258) for processing by GEO2R and incorporation into this study^14^. The data studied the comparison of expression of various human genes at different liver fibrosis stages in HCV-infected individuals^14^. Differentially expressed genes were screened from the GEO dataset with a threshold of |log2FC| > 1 and adjusted P < 0.02.

## Supplementary Information

Supplementary Information.

Supplementary Figure 1.

## Data Availability

The datasets generated and/or analyzed during the current study are available in the Gene Expression Omnibus (GEO) database, GSE33258.

## Abbreviations

ALP: Alkaline phosphatase
ALT: Alanine aminotransferase
APRI: Aspartate aminotransferase to platelet ratio index
AST: Aspartate aminotransferase
ATX: Autotaxin
AUC: Area under the receiver operating characteristic curve
DAA: Direct-acting antiviral
ECM: Extracellular matrix
FIB-4index: Fibrosis-4 index
GGTP: Gamma-glutamyl transpeptidase
HCC: Hepatocellular carcinoma
HCV: Hepatitis C virus
IQR: Interquartile range
NAFLD: Nonalcoholic fatty liver disease
NPV: Negative predictive value
PPV: Positive predictive value
TGF-β: Transforming growth factor-β
TSP2: Thrombospondin 2
