# Supplementary material for: Circulating thrombospondin 2 levels reflect fibrosis severity and disease activity in HCV-infected patients
Source: Sci Rep. 2022 Nov 7;12:18900. doi: 10.1038/s41598-022-23357-9 (PMC9640666; doi:10.1038/s41598-022-23357-9)
Supplement: Supplementary file 1 — Supplementary Information. [file 41598_2022_23357_MOESM1_ESM.docx]

**Supplementary Table 1. Clinicopathological features of patients with HCV in the validation cohort**

|  | All (N=80) |
| --- | --- |
|  | Median (IQR)/N (%) |
| Age (years) | 60 (51-65) |
| Male | 44 (55%) |
| Body mass index (kg/m^2^) | 23.5 (21.1-25.9) |
|  |  |
| **Laboratory data** |  |
| Albumin (mg/dL) | 4.3 (4.2-4.5) |
| Total bilirubin (mg/dL) | 0.80 (0.60-1.00) |
| Direct bilirubin (mg/dL) | 0.15 (0.10-0.28) |
| AST (U/L) | 66 (42-103) |
| ALT (U/L) | 89 (54-128) |
| ALP (U/L) | 286 (239-371) |
| GGTP (U/L) | 52 (32-89) |
| Total cholesterol (mg/dL) | 169 (150-185) |
| Triglycerides (mg/dL) | 98 (74-136) |
| Alpha-fetoprotein (ng/mL) | 8.2 (3.7-15.3) |
| PT (%) | 98.0 (89.5-106.7) |
| TSP2 (ng/mL) | 41.2 (27.7-61.2) |
| ATX (mg/L) | 1.8 (1.2-2.3) |
| FIB-4 index | 3.29 (1.78-4.73) |
| Forn’s index | 6.72 (5.33-7.94) |
| APRI | 1.8 (1.0-3.0) |
| Platelet count (×10^4^/uL) | 14.0 (11.0-17.7) |
|  |  |
| **HCV** |  |
| Genotype (1/2) | 54/26 |
|  |  |
| **Pathology** |  |
| METAVIR |  |
| Fibrosis stage (F0-1/F2/F3-4) | 31/21/28 |
| Activity grade (A0/A1/A2/A3) | 9/28/37/6 |

ALT: alanine aminotransferase, ALP: alkaline phosphatase, APRI: aspartate aminotransferase to platelet ratio index, AST: aspartate aminotransferase, ATX: autotaxin, FIB-4 index: fibrosis-4 index, GGTP: gamma-glutamyl transpeptidase, HCV: hepatitis C virus, IQR: interquartile range, PT: prothrombin time, TSP2: thrombospondin 2

**Supplementary Table 2. Correlation between TSP2 and clinicopathological parameters in the discovery cohort**

|  | Correlation with TSP2  (N=80) | |
| --- | --- | --- |
|  | *r* | P value |
| Age | -0.041 | 0.7189 |
| Body mass index | 0.079 | 0.2734 |
|  |  |  |
| **Laboratory data** |  |  |
| Albumin | -0.274 | **0.0168** |
| Total bilirubin | 0.115 | 0.3223 |
| Direct bilirubin | 0.404 | **0.0048** |
| AST | 0.488 | **<0.0001** |
| ALT | 0.479 | **<0.0001** |
| ALP | 0.337 | **0.0029** |
| GGTP | 0.521 | **<0.0001** |
| Total cholesterol | 0.030 | 0.8157 |
| Triglycerides | 0.199 | 0.1460 |
| Alpha-fetoprotein | 0.657 | **<0.0001** |
| PT | -0.161 | 0.2700 |
| ATX | 0.511 | **<0.0001** |
| FIB-4 index | 0.374 | **0.0007** |
| Forn’s index | 0.379 | **0.0007** |
| APRI | 0.460 | **<0.0001** |
| Platelet count | -0.357 | **0.0014** |
|  |  |  |
| **Pathology** |  |  |
| METAVIR |  |  |
| Fibrosis stage | 0.465 | **0.0001** |
| Activity grade | 0.449 | **0.0003** |

Correlations were calculated using Spearman’s test.

ALT: alanine aminotransferase, ALP: alkaline phosphatase, APRI: aspartate aminotransferase to platelet ratio index, AST: aspartate aminotransferase, ATX: autotaxin, FIB-4 index: fibrosis-4 index, GGTP: gamma-glutamyl transpeptidase, PT: prothrombin time, TSP2: thrombospondin 2

**Supplementary Table 3. Diagnostic performance of TSP2 and other clinical parameters for predicting advanced fibrosis stage (≥F3) and activity grade (≥A2) in patients with HCV in the validation cohort**

| **≥F3** | Cut-off  value | AUC | Sensitivity (%) | Specificity (%) | PPV  (%) | NPV  (%) |
| --- | --- | --- | --- | --- | --- | --- |
| TSP2 | 50.3 (ng/mL) | 0.82 | 78.8 | 68.0 | 82.0 | 63.3 |
| ATX | 2.23 (mg/L) | 0.72 | 78.8 | 50.0 | 74.5 | 56.0 |
| FIB-4 index | 4.3 | 0.79 | 78.4 | 56.0 | 77.4 | 60.0 |
| Forn’s index | 6.9 | 0.78 | 76.0 | 66.7 | 80.9 | 60.1 |
| APRI | 2.1 | 0.75 | 68.6 | 66.7 | 79.5 | 52.9 |
| Platelet count | 12.0 (×10^4^/uL) | 0.79 | 78.4 | 59.7 | 78.4 | 59.3 |
|  |  |  |  |  |  |  |
| **≥A2** | Cut-off  value | AUC | Sensitivity (%) | Specificity (%) | PPV  (%) | NPV  (%) |
| TSP2 | 47.8 (ng/mL) | 0.79 | 83.8 | 60.5 | 64.5 | 81.2 |
| ATX | 1.90 (mg/L) | 0.66 | 67.6 | 48.9 | 53.2 | 63.6 |
| FIB-4 index | 3.5 | 0.66 | 56.8 | 53.7 | 53.8 | 58.9 |
| Forn’s index | 7.0 | 0.60 | 72.2 | 58.5 | 48.0 | 63.0 |
| APRI | 2.1 | 0.73 | 75.7 | 56.1 | 60.8 | 71.9 |
| Platelet count | 13.5 (×10^4^/uL) | 0.59 | 62.2 | 48.8 | 51.1 | 58.1 |

APRI: aspartate aminotransferase to platelet ratio index, ATX: autotaxin, FIB-4 index: fibrosis-4 index, TSP2: thrombospondin 2

**Supplementary Figure legends**

**Supplementary Figure 1. Summary of the article**

Serum TSP2 levels were moderately correlated with both liver fibrosis stage and activity grade. Moreover, comprehensive liver genetic analysis of HCV-infected patients confirmed that the expression of the *THBS2* gene encoding TSP2 was significantly higher in severely fibrotic F4 than in F1 patients.
