# Supplementary figures and images for: Circulating thrombospondin 2 levels reflect fibrosis severity and disease activity in HCV-infected patients
Source: Sci Rep. 2022 Nov 7;12:18900. doi: 10.1038/s41598-022-23357-9 (PMC9640666; doi:10.1038/s41598-022-23357-9)

## Slide 1
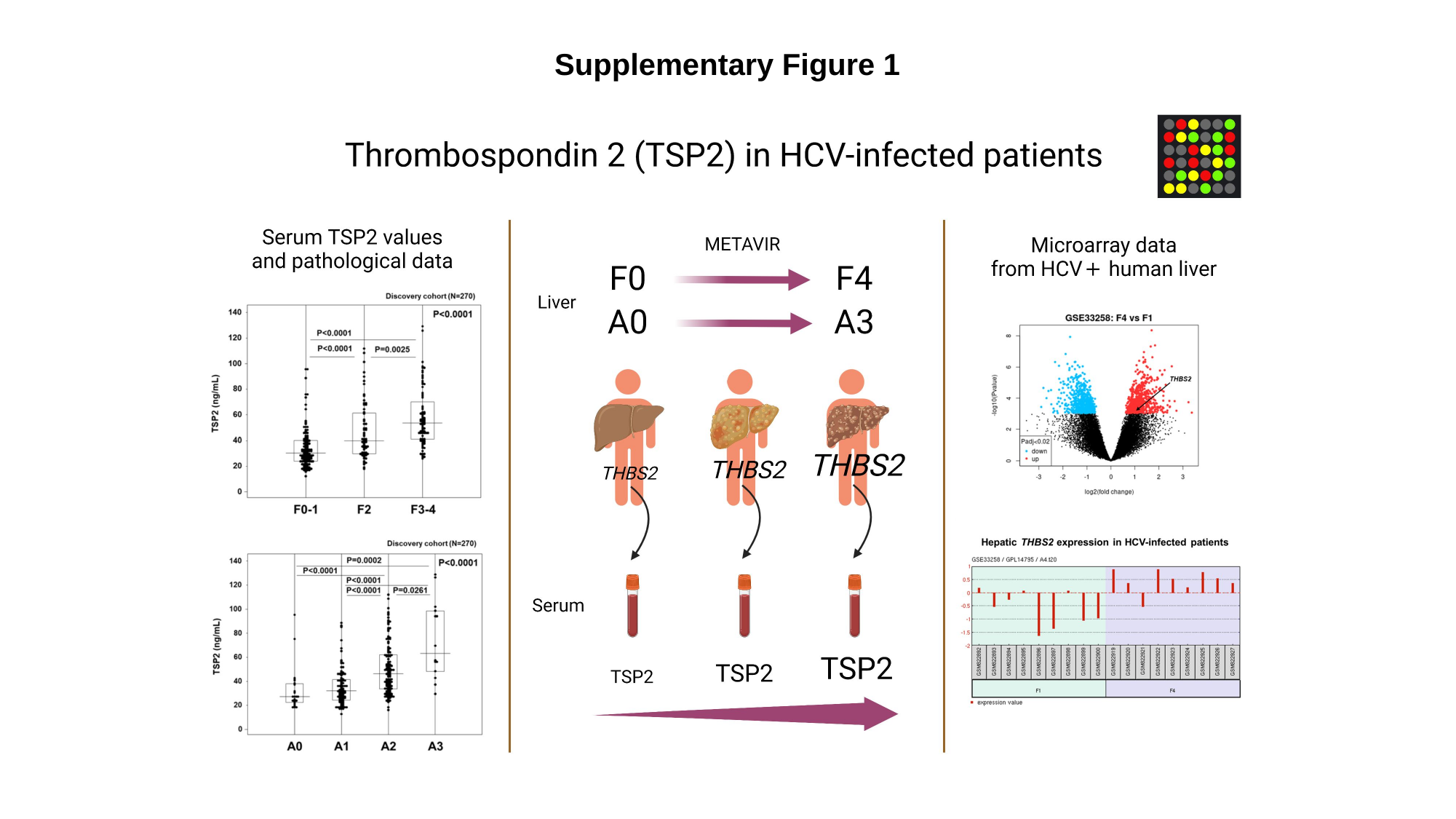

Supplementary Figure 1

Supplement: Supplementary file 2 — Supplementary Figure 1. [file 41598_2022_23357_MOESM2_ESM.pptx]
